# IL‐27: A Double‐Edged Sword in Immune Regulation via JAK‐STAT Orchestration

**DOI:** 10.1155/jimr/6176816

**Published:** 2026-06-02

**Authors:** Xiangqi Zhang, Chen Feng, Ye Xu, Yingqi Zeng, Qiulin Luo, Jiajie Qin, Tengfang Li, Longkai Peng, Hedong Zhang, Helong Dai

**Affiliations:** ^1^ Department of Immunology, School of Basic Medical Science, Central South University, Changsha, Hunan, China, csu.edu.cn; ^2^ Department of Kidney Transplantation, Center of Organ Transplantation, The Second Xiangya Hospital of Central South University, Changsha, Hunan, China, csu.edu.cn; ^3^ Center of Organ Transplantation, Xiangya Hospital, Central South University, Changsha, Hunan, China, csu.edu.cn; ^4^ Medical College of Guangxi University, Nanning, Guangxi, China, gxtcm.com

**Keywords:** IL-27, immune regulation, inflammation, the JAK-STAT signaling pathway

## Abstract

Interleukin‐27 (IL‐27) is a multifunctional cytokine produced mainly by innate immune cells that regulates immune responses primarily through the Janus kinase (JAK)‐signal transducer and activator of transcription (STAT) signaling pathway. The role of IL‐27 in immune regulation is complex and often paradoxical, with both pro‐inflammatory and immunosuppressive functions described, depending on the specific immune context and cellular milieu. Here, we systematically discuss the dual regulatory roles of IL‐27 in immune regulation, with a particular emphasis on its role in the JAK‐STAT signaling pathway. We highlight the latest advancements in understanding the functional differences and molecular mechanisms of IL‐27 in both adaptive and innate immune cells. We further elaborate on IL‐27‐mediated immune regulation through nonimmune cells. Understanding the multifaceted and context‐dependent properties of IL‐27 is essential for deciphering the precise role of IL‐27 in health and disease and for developing targeted immunotherapies. This review systematically analyzes the “double‐edged sword” mechanism of IL‐27, providing a theoretical and translational basis for targeting the JAK‐STAT pathway.

## 1. Introduction

Intercellular communication via diverse signaling systems is essential for immune homeostasis, with interleukin‐27 (IL‐27) serving as a paramount regulator. Composed of p28 and Epstein–Barr virus‐induced gene 3 (EBI3) subunits, IL‐27 is primarily secreted by innate immune cells [1]. It signals through a heterodimeric receptor (IL‐27Rα and gp130), where IL‐27Rα provides the specificity required to initiate its complex downstream effects through the JAK‐STAT pathway [2].

Upon binding of IL‐27 to its receptor, various signaling cascades are activated, primarily through the JAK‐STAT signaling pathway, thereby altering the behavior of target cells [3]. IL‐27 signaling in target cells is primarily mediated by phosphorylated STAT1 (pSTAT1) and pSTAT3, which serve as the key functional effectors. Within this signaling framework, pSTAT3 acts as the primary driver for transcription as STAT3‐deficient cells show the least gene induction, whereas pSTAT1 defines the cytokine‐specific response [4]. During this process, corresponding negative feedback mechanisms, such as suppressor of cytokine signaling (SOCS) protein‐mediated negative regulation, are also initiated within the body to prevent excessive immune responses from causing tissue damage.

A prominent and well‐known characteristic of IL‐27 is its duality: it can both promote inflammation and suppress immune reactions. This duality is not an intrinsic property of the cytokine itself but rather is dependent on the specific immune context. On the basis of the stimuli provided by the surrounding environment, IL‐27 precisely regulates the intensity and direction of the immune system to eliminate threats with minimal collateral damage and restore internal environmental balance, thereby providing a survival advantage to the organism.

This review focuses on IL‐27 and its role in the JAK‐STAT signaling pathway, systematically organizing its dual regulatory roles within the immune system. It places particular emphasis on discussing the functional differences and molecular mechanisms of IL‐27 via the JAK‐STAT pathway in adaptive and innate immune cells, as well as its crucial role in the development of inflammation and disease.

## 2. Structure, Biological Functions, and Signal Transduction Characteristics of IL‐27

### 2.1. IL‐27 Cytokine Structure

**Figure 1:**
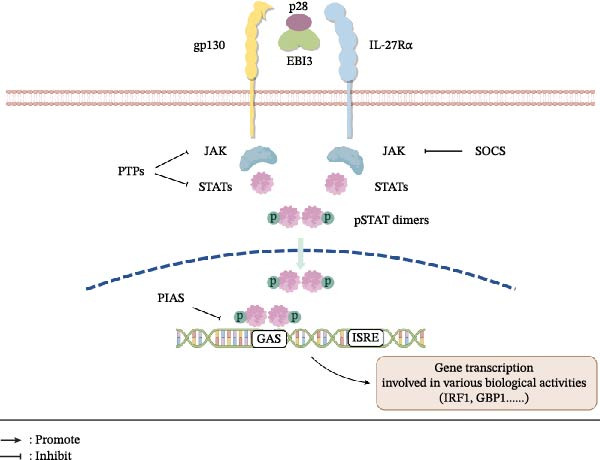
Schematic diagram of the IL‐27 signaling pathway. IL‐27 is a heterodimeric cytokine composed of the p28 and EBI3 (Epstein–Barr virus‐induced gene 3) subunits. It signals by binding to a receptor complex on the cell membrane, which consists of gp130 (glycoprotein 130) and IL‐27Rα. This binding activates the Janus kinase (JAK)‐signal transducer and activator of transcription (STAT) pathway, leading to the phosphorylation of the downstream proteins STATs. Phosphorylated STAT (pSTAT) dimers are translocated into the nucleus, where they bind to the ISRE (interferon‐stimulated regulatory element) and GAS (gamma‐activated sequence) DNA response elements, respectively. This initiates the transcription of various target genes, such as GBP1 (guanylate‐binding protein 1) and IRF1 (interferon regulatory factor 1), which are involved in a wide range of biological activities. Moreover, this pathway is strongly regulated by several negative regulatory proteins, including SOCS (suppressor of cytokine signaling) family proteins, PTP (protein tyrosine phosphatase), and PIAS (protein inhibitor of activated STAT) family proteins (created with Figdraw, https://www.figdraw.com).

IL‐27 was first reported in 2002 as a unique heterodimeric cytokine composed of p28 (a four‐helix bundle subunit) and the soluble cytokine receptor‐like protein EBI3 (Figure 1) [1]. Independent of EBI3, p28 can be secreted on its own and bind to gp130, thereby antagonizing IL‐6/IL‐27‐mediated STAT1/3 phosphorylation. This action leads to a marked increase in activated splenic T cells, antagonizes the development of Th17 cells, and causes defective thymus‐dependent B‐cell responses [5]. This intrinsic capacity of the p28 subunit to function antagonistically, in contrast to the signaling activation of the full heterodimer, serves, to a certain extent, as a molecular microcosm of the broader ‘double‐edged sword’ nature of IL‐27. It establishes an immediate precedent for the cytokine’s complex biology, demonstrating that opposing functions are embedded even within its structural components. Despite these independent functions, the full signaling activity of IL‐27 still requires the synergistic interaction of p28 with EBI3. The formation of a highly specific and stable noncovalent complex between the p28 and EBI3 subunits is the fundamental basis and structural guarantee for the biological functions of IL‐27.

### 2.2. IL‐27 Receptor Complex and Ligand Binding

IL‐27 signal transduction relies on its receptor complex, which is composed of two subunits, gp130 and IL‐27Rα (also known as TCCR or WSX‐1) (Figure 1). In 2004, research confirmed that the functional IL‐27 receptor is composed of IL‐27Rα and gp130, with the former primarily mediating STAT1 phosphorylation and the latter activating STAT3 [2]. Both subunits are coexpressed in various immune cells and certain nonimmune cells, including monocytes, dendritic cells (DCs), T cells, B cells, natural killer (NK) cells, mast cells, endothelial cells, hepatocytes, cardiac Sca‐1+ cells, and renal tubular epithelial cells [2, 6–8].

The structural assembly mechanism of the IL‐27 receptor signaling complex involves primarily IL‐27, which forms a stable tetrameric structure by bridging IL‐27Rα and gp130. Specifically, the p28 subunit acts as the core driving component, preferentially binding to the hinge region of IL‐27Rα. Through its D2 domain, EBI3 forms a stem–stem contact with the D2 domain of IL‐27Rα, further stabilizing ligand‒receptor binding. The D1 Ig domain of gp130 subsequently interacts with both p28 and EBI3 to complete the ternary assembly of the signaling complex [9]. In this multipoint cooperative binding model, p28 predominantly drives the formation of the complex, whereas EBI3 plays a crucial auxiliary role in stabilizing the structure and facilitating signal transduction. Crucially, this tetrameric assembly imposes a geometric constraint, facilitating the trans‐phosphorylation of associated JAKs. This structural arrangement dictates the asymmetric signaling bias, preferentially recruiting STAT1 via IL‐27Rα and STAT3 via gp130 to link structure to specific functional outcomes [2].

### 2.3. Cellular Sources and Regulation of IL‐27 Expression

Although IL‐27 is predominantly produced by antigen‐presenting cells, its cellular source is not static; rather, it is dynamically regulated by the specific immune context and disease stage to dictate functional outcomes. In the early phase of immune challenges, innate sentinel cells such as DCs and macrophages serve as the primary sources. For instance, during acute dengue virus infection, DCs rapidly secrete IL‐27, which is critical for driving the initial generation of follicular T helper (Tfh) cells [10]. Similarly, monocytes and macrophages produce IL‐27 in response to bacterial stimuli like lipopolysaccharide (LPS), thereby initiating early inflammatory control [11].

As the immune response progresses, the principal source of IL‐27 can shift from innate to adaptive immune cells. In the context of persistent lymphocytic choriomeningitis virus clone 13 infection, B cells emerge as a pivotal source. Unlike the early DC‐driven response, B cell‐derived IL‐27 is essential for promoting the survival of virus‐specific CD4^+^ T cells and sustaining Tfh function during the chronic phase [12]. This transition highlights a staged functional complementarity between DC‐derived and B cell‐derived IL‐27 in orchestrating immunity. Beyond these principal sources, myeloid‐derived suppressor cells, T cells, and tissue‐resident cells such as osteoclasts and osteoblasts can also be induced to secrete IL‐27 within specific microenvironments [13–15].

The induction of IL‐27 expression is initiated by signals such as pathogen‐associated molecular patterns (PAMPs) or specific cytokines such as interferon‐gamma (IFN‐γ), which activate pattern recognition receptors or interferon receptors on the surface of antigen‐presenting cells. Toll‐like receptors (TLRs) play a critical role in this initial activation [16]. In activated target cells, a series of intracellular signaling pathways, including the MyD88‐dependent pathway and the TRIF‐dependent pathway, are triggered [17]. Various transcription factors, including IRF1, IRF3, IRF8, IRF9, and NF‐κB, subsequently bind to the promoter regions of the *IL27p28* and *EBI3* genes, thereby upregulating their expression [18–21].

Upon binding to its receptor, IL‐27 activates a cascade of signaling pathways to exert diverse biological effects, with the major pathways being the JAK‐STAT signaling pathway and the mitogen‐activated protein kinase (MAPK) signaling pathway [3]. This review focuses primarily on the role of the JAK‐STAT signaling pathway in IL‐27‐mediated biological effects.

## 3. The JAK‐STAT Pathway in IL‐27 Signaling

### 3.1. Core Components: JAKs and STATs

The JAK‐STAT cascade is the main signaling mechanism of IL‐27, which includes four Janus kinases (JAK1‐3, TYK2) and seven STAT members [22, 23]. The IL‐27 signaling is mediated by the heterodimeric receptor IL‐27Rα and gp130, which recruit JAK1, JAK2, and TYK2. Specifically, IL‐27Rα binds to JAK kinases through Box1, whereas gp130 binds to Box1 and Box2 [24]. These kinases then activate STAT1 and STAT3 as the main effectors, while STAT5 plays a context‐dependent role. This specific combination determines the unique immune regulatory characteristics of IL‐27 compared to other gp130 family cytokines. In murine naïve CD4^+^ T cells, both IL‐27 and IL‐6 were found to induce STAT1‐STAT3 and STAT3‐STAT3 dimers; however, the formation of STAT1‐STAT1 homodimers was unique to IL‐27 stimulation [4]. This differential dimer composition provides a mechanistic explanation for the STAT1‐driven specificity of IL‐27.

### 3.2. The JAK‐STAT Signaling Pathway: Canonical Activation and Regulation

#### 3.2.1. Activation Pathway

Upon IL‐27 binding, receptor‐associated JAKs phosphorylate cytoplasmic STATs. Of note, the basal pool of total STAT1 and STAT3 does not possess transcriptional activity. Only phosphorylated STATs can enter the nucleus to drive subsequent transcription [25]. In this process, canonical activation relies on tyrosine phosphorylation (e.g., Tyr705 on STAT3) to drive dimerization and nuclear entry [23, 26]. In contrast, serine phosphorylation (e.g., Ser727) provides a layer of fine‐tuned regulation, recruiting transcriptional co‐activators to ensure maximal transcriptional activity or modulating mitochondrial function [23, 27]. Across distinct cell subsets and varying disease states, microenvironmental differences significantly shift the relative phosphorylation ratio between different STAT members (such as STAT1 and STAT3) [25]. At the molecular level, this dynamic phosphorylation balance reveals the underlying mechanism by which IL‐27 exerts its “double‐edged sword” immunoregulatory effects through the JAK‐STAT pathway. Once in the nucleus, pSTAT dimers bind to specific DNA cis‐acting elements (e.g., gamma‐interferon activated sequences [GASs] or interferon‐stimulated regulatory elements [ISREs]) to initiate gene transcription (Figure 1). Notably, STAT1 chromatin binding largely depends on STAT3, highlighting a cooperative model in which STAT1 refines the STAT3‐mediated transcriptional program to confer IL‐27 specificity [4]. Different combinations of STAT proteins can activate distinct DNA elements, thereby regulating gene transcription programs required for diverse physiological or immune responses. For instance, STAT1 homodimers recognize GAS elements, which are activated by IFN‐γ signaling to promote the expression of immunoregulatory and inflammatory genes (e.g., IRF1 and GBP1) [28, 29] (Figure 1).

Notably, multiple JAK inhibitors (JAKinibs) have been widely used globally in recent years to mitigate pathological processes driven by dysregulated signaling [25]. For example, baricitinib (a selective JAK1/2 inhibitor) effectively promotes hair regrowth in patients with severe alopecia areata [30]. Furthermore, novel agents such as deucravacitinib selectively inhibits TYK2 via an allosteric mechanism, offering a superior safety profile in psoriasis treatment [31]. Although specific clinical trials targeting IL‐27 blockade are currently in the early stages, given that IL‐27 signaling strictly relies on JAK1, JAK2, and TYK2, these existing pharmacologic agents possess the intrinsic capacity to modulate the IL‐27 pathway.

#### 3.2.2. Negative Feedback Mechanisms

The JAK‐STAT signaling pathway is tightly regulated by several negative feedback mechanisms that ensure signal attenuation and prevent pathological activation. Key regulators include the SOCS family, which inhibits JAK activity and promotes proteasomal degradation of signaling components; protein inhibitors of activated STATs (PIAS), which block STAT DNA binding and recruit transcriptional repressors; and protein tyrosine phosphatases (PTPs), such as SHP‐1, SHP‐2, and TC‐PTP, which dephosphorylate JAKs and STATs to terminate signaling [25]. These mechanisms form an integrated negative feedback loop crucial for maintaining immune homeostasis and preventing chronic inflammation or oncogenic transformation.

## 4. Dual Regulation of Adaptive Immune Cells

### 4.1. CD4^+^ T Cells

IL‐27 activates multiple components of the JAK‐STAT signaling pathway, including JAK1, JAK2, TYK2, STAT1, STAT2, STAT3, and STAT5, in naïve CD4^+^ T cells. Among these, STAT1 plays a critical role in the IL‐27‐induced expression of T‐bet, the subsequent IL‐12Rβ2, and MHC class I, whereas STAT3 is closely associated with IL‐27‐induced proliferation [32, 33].

In mature CD4^+^ T cells, IL‐27 mainly activates STAT1 and STAT3. The IL‐27/STAT3 axis can suppress the expression of certain cytokines, including IL‐2, IL‐4, IL‐17, and TNF‐α [33]. However, in a mouse model of allogeneic skin transplantation, IL‐27 promoted the infiltration and proliferation of CD4^+^ T cells within the graft and inhibited their apoptosis via the STAT1/3/5 signaling pathway, leading to rejection [34]. The serum of recipient mice contained high levels of IFN‐γ and IL‐27 but low levels of IL‐10 [34].

In CD4^+^ T cells, IL‐27‐activated STAT1 and STAT3 play distinct yet cooperative roles. STAT3 is the primary driver of transcription, as STAT3‐deficient cells exhibit minimal gene induction. In contrast, STAT1 defines cytokine‐specific responses: while STAT1‐deficient cells still respond to IL‐27, their gene expression profiles mirror those induced by IL‐6, indicating a loss of specificity. This role is further supported by STAT1 gain‐of‐function mutations in patients, which lead to aberrant immune gene regulation.

#### 4.1.1. Th1 Cells

During the differentiation of Th1 cells, by activating multiple key molecules in the JAK‐STAT pathway, IL‐27 has both pro‐inflammatory and anti‐inflammatory effects. Its core induction mechanisms include the STAT1/T‐bet, STAT1/intercellular adhesion molecule‐1 (ICAM‐1)/lymphocyte function‐associated antigen‐1 (LFA‐1)/extracellular signal‐regulated kinases 1 and 2 (ERK1/2), and p38 MAPK/T‐bet pathways [35, 36].

**Figure 2:**
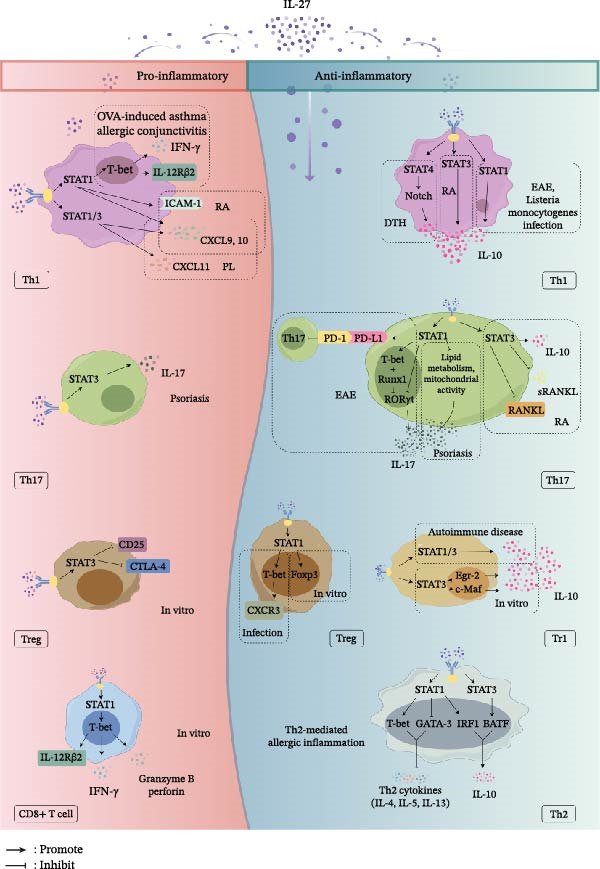
Dual pro‐ and anti‐inflammatory roles of IL‐27 in T‐cell subsets via the JAK‐STAT pathway. This diagram highlights the JAK‐STAT signaling pathway as a critical intracellular hub that dictates whether T‐cell subsets perform pro‐inflammatory or anti‐inflammatory functions depending on the specific microenvironment. The left panel (red) depicts its pro‐inflammatory roles, while the right panel (blue) shows its anti‐inflammatory roles. The pro‐inflammatory mechanisms of the IL‐27/STAT axis include enhancing Th1 cell differentiation, stimulating Th17‐mediated inflammation in specific contexts, inhibiting Treg development, and augmenting CD8^+^ T‐cell cytotoxicity. Conversely, its anti‐inflammatory mechanisms involve stimulating IL‐10 secretion from Th1 cells, suppressing the differentiation of Th17 and Th2 cells, promoting Treg cell function, and inducing the generation of Tr1 cells (created with Figdraw, https://www.figdraw.com). OVA: ovalbumin; IFN: interferon; IL: interleukin; STAT: signal transducer and activator of transcription; ICAM‐1: intercellular adhesion molecule‐1; RA: rheumatoid arthritis; CXCL: chemokine (C‐X‐C motif) ligand; PL: preterm labor; CTLA‐4: cytotoxic T‐lymphocyte‐associated protein; Treg: T regulatory; DTH: delayed‐type hypersensitivity; EAE: experimental autoimmune encephalomyelitis; PD‐1: programmed death‐1; PD‐L1: programmed death‐ligand 1; RANKL: receptor activator of nuclear factor‐κB ligand; CXCR3: chemokine receptor 3; Tr1: type 1 regulatory T; GATA‐3: GATA binding protein 3; IRF1: interferon regulatory factor 1; BATF: basic leucine zipper ATF‐like transcription factor.

Th1‐type pro‐inflammatory effects mediated by IL‐27 have been confirmed in various inflammatory diseases. In an ovalbumin (OVA)‐induced asthma mouse model, intranasal administration of IL‐27 upregulated the expression of STAT1 and T‐bet and remodeled the local Th1‐type immune environment to indirectly inhibit Th2‐associated allergic pneumonia and remodeling [37]. In a model of allergic conjunctivitis, the absence of IL‐27 signaling leads to impaired Th1 responses, characterized by significantly reduced expression levels of IFN‐γ and T‐bet [38]. Within the IL‐27/STAT1/T‐bet pathway, induced T‐bet can further upregulate the expression of IL‐12Rβ2, enabling T cells to respond to IL‐12 and laying the molecular foundation for subsequent Th1 cell maturation and functional consolidation [39, 40] (Figure 2). Furthermore, in the context of HIV infection, IL‐27 activates the STAT1/T‐bet signaling pathway in TIGIT+HIV‐Gag‐specific CD4^+^ T cells, promoting the differentiation of these dysfunctional T cells toward a Th1 phenotype while inhibiting Th2 and Th17 differentiation, thereby enhancing the immune capacity of people living with HIV (PLWH) [39].

Second, the STAT1/ICAM‐1/LFA‐1/ERK1/2 axis plays a pro‐inflammatory role: the IL‐27/STAT1 axis upregulates intercellular adhesion molecule‐1 (ICAM‐1) on the cell surface, and the physical binding of ICAM‐1 to lymphocyte function‐associated antigen‐1 (LFA‐1) on another cell’s surface can activate ERK1/2, ultimately promoting Th1 differentiation [36]. In the inflamed synovial tissue of patients with rheumatoid arthritis (RA), IL‐27 levels are significantly elevated [41]. Increased levels of IL‐27 activate the STAT1 signaling pathway and increase the secretion of chemokines (CXCL9 and CXCL10) and the expression of surface adhesion molecules (ICAM‐1), thereby promoting the intensity of the Th1‐type immune environment in the synovium of joints [42]. Furthermore, the IL‐27‐induced JAK2/STAT1/STAT3 and IFN‐γ/ERK signaling pathways can promote the expression of inflammatory mediators (including CXCL9, CXCL10, and CXCL11) in fetal membrane tissues, inducing the infiltration of Th1 cells to the maternal‐fetal interface and thereby increasing the risk of preterm birth through a pro‐inflammatory effect [43, 44] (Figure 2).

Notably, compared with IL‐6, which shares the same signal‐transducing subunit (gp130), IL‐27 induces more sustained STAT1 phosphorylation in Th1 cells and upregulates the transcription factor IRF1 [45]. The synergistic effect of pSTAT1 and IRF1 further amplifies IL‐27‐specific interferon‐like gene expression and protein remodeling [45]. This mechanism is supported in T cells from patients with systemic lupus erythematosus (SLE), where high intracellular levels of STAT1 protein further increase the IL‐27/STAT1‐mediated pro‐inflammatory effects [45].

Conversely, concerning its anti‐inflammatory effects, IL‐27 induces STAT4 signaling in synergy with the Notch signaling pathway, and the IL‐27/STAT3 axis can stimulate Th1 cells to secrete IL‐10 [46, 47]. In disease models of experimental autoimmune encephalomyelitis (EAE) and *Listeria monocytogenes* infection, the IL‐27/STAT1 axis promotes the differentiation of CD4^+^ T cells into IL‐10+IFN‐γ+Foxp3‐ Th1 cells [48]. The production of IL‐10 can suppress the overactivation of other surrounding immune cells, forming a negative feedback loop that limits excessive inflammatory damage and protects host tissues (Figure 2).

Overall, the net inflammatory outcome of IL‐27 signaling in Th1 cells depends largely on the dominance of STAT1‐driven pathways (which favor Th1 polarization and IFN‐γ production) versus STAT3/STAT4‐mediated signaling (which promotes IL‐10 induction). High‐intensity or sustained STAT1 activation—often seen in strong inflammatory milieus enriched with IL‐12, type I interferons, or high gp130/STAT1 availability—biases IL‐27 toward a pro‐inflammatory effect, whereas environments that favor STAT3/STAT4 activation, such as chronic or resolving inflammation, preferentially shift IL‐27 toward an anti‐inflammatory IL‐10–producing Th1 phenotype.

#### 4.1.2. Th2 Cells

IL‐27 simultaneously activates STAT1 and STAT3 in Th2 cells. Here, the IL‐27/STAT1 axis acts as the primary driver for inhibiting Th2 differentiation by upregulating T‐bet and downregulating GATA binding protein 3 (GATA‐3), thereby suppressing Th2 cytokine production [38, 49, 50]. Given that Th2 cells play a dominant role in the pathogenesis of allergic asthma, the following section focuses on the context of asthma to elaborate on the complex role of the IL‐27/STAT axis in regulating the Th2 immune response.

The IL‐27/STAT1/GATA‐3 signaling pathway reduces Th2 cytokines (including IL‐4, IL‐5, and IL‐13) and suppresses the disease course of asthma, with STAT3 playing an auxiliary role [37]. Furthermore, IL‐27 can indirectly improve the pulmonary Th2‐type inflammatory environment by promoting the differentiation of type 1 regulatory T (Tr1) cells, which can produce IL‐10, via the STAT1/IRF1 and STAT3/BATF axes [51]. Notably, this mechanism is effective in preventative models where IL‐27 administration increases lung Tr1 frequency and reduces Th2 cytokines; however, IL‐27 fails to alleviate the condition when administered therapeutically in established asthma [52].

This lack of therapeutic efficacy is likely due to the established inflammatory microenvironment interfering with IL‐27 signaling. In the in vivo microenvironment, high levels of IL‐4 form a signaling axis with SOCS3, which inhibits the suppressive effect of the IL‐27/STAT1‐mediated pathway on Th2 differentiation, explaining the persistent Th2 immune response [50]. Additionally, in vitro experiments utilizing human bronchial epithelial cells (HBECs) revealed that costimulation with IL‐27 and IL‐13 (a key type 2 cytokine) selectively activated STAT1 while attenuating STAT3 expression [53]. This skewed signaling promoted the expression of CXCL9, thereby exacerbating the inflammatory response rather than suppressing it [53]. These findings suggest that therapeutic targeting of IL‐27 in asthma may require combinatorial strategies to first neutralize these interfering cytokines or bypass these inhibitory feedback loops.

#### 4.1.3. Th17 Cells

In various disease models, such as those for autoimmune and infectious diseases, the primary effect of the IL‐27/STAT axis in Th17 cells is the inhibition of their differentiation and function, thereby exerting an anti‐inflammatory effect (Figure 2).

First, IL‐27 can directly or indirectly block the production of the master transcription factor for Th17 cells, RORγt, thus impeding Th17 cell differentiation at its source. In EAE and delayed‐type hypersensitivity, IL‐27‐activated STAT1 can directly inhibit the expression and function of the key Th17 transcription factor RORγt, consequently downregulating the production of IL‐17A [38, 54]. As demonstrated in in vivo models such as EAE and through in vitro T‐cell experiments, the physical binding of T‐bet—induced by the IL‐27/STAT1 axis—to the transcription factor Runx1 indirectly inhibits RORγt production, thereby initiating the Th1 differentiation program while concurrently shutting down the Th17 differentiation pathway [55, 56].

Second, in addition to inhibiting differentiation, IL‐27 can directly attenuate the pathological damage caused by the Th17‐type inflammatory environment through other mechanisms. IL‐27 upregulates programmed death‐ligand 1 (PD‐L1) expression on naïve T cells via STAT1; the subsequent binding of PD‐L1 to PD‐1 inhibits Th17 cell differentiation and alleviates the pathological progression of EAE [57].

In patients with RA, the IL‐27/STAT3 axis can reduce the expression of receptor activator of nuclear factor‐κB ligand (RANKL) on the surface of naïve CD4^+^ T cells and the secretion of soluble RANKL (sRANKL), which in turn inhibits inflammatory damage, such as bone erosion mediated by Th17 cells [47]. The IL‐27/STAT1 axis can also inhibit Th17 cell differentiation in RA patients, a mechanism that is counteracted by the IL‐6/STAT3 axis [58].

In addition to its effects on autoimmune diseases, the inhibitory effect of the IL‐27/STAT axis on Th17 cells has been verified in certain infectious diseases. In a mouse model of visceral leishmaniasis, the IL‐27/STAT1/3 axis inhibited the secretion of IL‐17 by Th17 cells, thereby reducing the protective, IL‐17‐mediated inflammatory response of neutrophil recruitment and ultimately leading to weakened host resistance and disease exacerbation [59]. Furthermore, in vitro experiments have shown that the IL‐27/STAT3 axis can stimulate Th17 cells to secrete IL‐10, resulting in broad anti‐inflammatory effects [47].

Furthermore, we highlight another distinct population of IL‐17‐secreting innate immune cells: γδ T17 cells. A fundamental distinction between γδ T17 and conventional Th17 cells lies in the composition of their T‐cell receptor (TCR) chains. In a murine model of psoriasis, the IL‐27/STAT1/STAT3 axis exerts a dual regulatory effect on γδ T17 cells. Specifically, the IL‐27/STAT1 axis inhibits IL‐17 production by suppressing lipid synthesis metabolism and mitochondrial activity in γδ T17 cells, ultimately alleviating psoriasis‐like skin inflammation in mice [60]. Conversely, the IL‐27/STAT3 axis promotes the expression of IL‐17, although the specific mechanism remains unclear [60]. This molecular‐level signaling competition offers a plausible mechanistic framework that helps explain the apparently contradictory clinical observations of IL‐27 in psoriasis. The fact that the equilibrium between STAT1 and STAT3 signaling may shift during the course of the disease likely underlies the significant controversy in the current literature regarding the protective versus pathogenic effects of IL‐27. Shibata et al. [61] observed elevated serum IL‐27 levels in psoriasis patients, supporting a “pro‐inflammatory” role. Conversely, Chen et al. [62] support an “anti‐inflammatory” role, noting that IL‐27 levels are actually downregulated in patients with moderate‐to‐severe disease, and that IL‐27 can ameliorate disease severity by inhibiting IL‐17 and its metabolism (as seen in the aforementioned γδ T17 mechanism). More interestingly, EL‐Komy et al. [63] found a negative correlation between serum IL‐27 levels and disease severity (PASI score). This suggests that IL‐27 may be upregulated as a protective factor (dominated by STAT1) during the early stages of the disease, but as the pathology worsens, this protective mechanism may fail or be overwhelmed by STAT3‐mediated pro‐inflammatory effects.

#### 4.1.4. Tregs and Tr1 Cells

In vitro studies have shown that during the induced differentiation of iTregs, IL‐27 activates STAT1, which then binds to the *Foxp3* gene promoter [64]. This enhances epigenetic modifications, such as histone acetylation, thereby promoting *Foxp3* expression and increasing the immunosuppressive capacity of cells [64]. Conversely, IL‐27 can also inhibit TGF‐β‐induced iTreg differentiation via a STAT3‐dependent signaling pathway, a process accompanied by the downregulation of CD25 and cytotoxic T‐lymphocyte‐associated protein‐4 (CTLA‐4) expression on the iTreg cell surface [65, 66] (Figure 2). This dual regulation of Treg cells exhibits notable species specificity: in murine models, STAT3 signaling predominantly mediates the inhibition of Treg expansion, whereas in human Treg cells, IL‐27 appears to preferentially enhance their suppressive function via STAT1 signaling [8].

In various murine infection models, including those for *Toxoplasma gondii* and *Leishmania major*, IL‐27 can activate STAT1, STAT3, and STAT5 in both natural and induced Tregs [67, 68]. Specifically, the IL‐27/STAT1/T‐bet/chemokine receptor 3 (CXCR3) axis promotes the migration of these Treg cells to sites of Th1‐type inflammation, enabling them to suppress inflammatory damage [67, 68]. In contrast, while the TGF‐β/Smad3/*Foxp3* axis promotes the differentiation of CD4^+^ T cells into Foxp3+ Tregs, the IL‐27/STAT3 axis can inhibit this mechanism [69].

In a murine EAE model and in bone marrow chimaera models, the IL‐27/STAT1/STAT3 axis promotes the differentiation of Tr1 cells and their subsequent secretion of IL‐10, which in turn suppresses autoimmunity and tissue inflammation [70–72]. However, the IL‐27/metallothionein axis can also inhibit the differentiation of Tr1 cells [70]. In vitro experiments have demonstrated that the IL‐27/STAT3 axis can induce the expression of the transcription factor Egr‐2, which then binds to the promoter region of *Blimp-1* to drive the differentiation of naïve CD4^+^ T cells into Tr1 cells and stimulate their production of IL‐10 [73]. The IL‐27/c‐Maf axis is also essential for Tr1 differentiation: the transcription factor c‐Maf promotes the secretion of both IL‐10 and IL‐21 to sustain Tr1 growth, with STAT3 likely positioned upstream of c‐Maf in this pathway [74] (Figure 2).

### 4.2. CD8+ T Cells

By activating the STAT signaling pathway, predominantly via STAT1, IL‐27 exerts complex and context‐dependent bidirectional control over CD8+ T cells. This can manifest as either an augmentation of their effector functions to promote inflammation and pathogen clearance or, under specific conditions, the mediation of immunosuppression (Figure 2).

In numerous contexts, the IL‐27/STAT1 axis functions by increasing the cytotoxic capacity and pro‐inflammatory activity of CD8+ T cells. Indeed, the role of IL‐27 in enhancing the cytotoxic function of CD8+ T cells is extensively documented in the literature [75–78]. In vitro experiments revealed that within CD8+ T cells, the IL‐27/STAT1 axis induces not only *T-bet* but also the transcription factor EOMES. *T-bet* promotes the expression of IL‐12Rβ2, while *T-bet* and EOMES together promote the expression of granzyme B (GzmB) and a slight increase in perforin levels, ultimately enhancing the cytotoxicity and killing ability of CTLs [79, 80]. Notably, a study demonstrates that beyond classical cytotoxicity, GzmB also participates in extracellular matrix remodeling and exacerbates tissue injury in autoimmune diseases [81]. Furthermore, the regulation of cytotoxic machinery may involve a cooperative role of STAT3. Evidence from cytokine signaling studies indicates that STAT3 can govern the expression of critical effector molecules, including GzmB, perforin, and the signaling adaptor DAP10, to mediate the lytic function of cytotoxic lymphocytes [82]. Given that IL‐27 activates both STAT1 and STAT3, it is plausible that this dual signaling orchestrates a synergistic upregulation of these cytotoxic mediators.

IL‐27 is capable of potentiating the antitumor CD8+ T‐cell immune response through the synergistic activation of STAT1, 2, 3, 4, and 5, with STAT1 and STAT3 playing particularly pivotal roles [79]. Furthermore, research utilizing a nonobese diabetic (NOD) mouse model revealed that the IL‐27/STAT1 axis not only influences Th1 cells but also promotes the accumulation and activation of pro‐inflammatory CD8+ T cells within pancreatic islets, thereby exacerbating local inflammation [83].

During viral or protozoan infections, IL‐27 activates STAT1 via its cognate receptor, which in turn induces the expression of *T-bet*; *T-bet* ultimately drives the production of the pro‐inflammatory cytokine IFN‐γ by these CD8+ T cells [84]. In the context of lymphocytic choriomeningitis virus (LCMV) infection, the therapeutic antiviral effect of IL‐27 is also contingent upon the STAT1‐driven expression of interferon regulatory factor 1 (IRF1); the subsequent activation of IRF1 promotes the expansion of virus‐specific CXCR5+ CD8+ T cells, leading to effective control of viral replication [85]. Furthermore, in antiretroviral therapy (ART)‐treated PLWH, IL‐27 activates the STAT1/T‐bet pathway in TIGIT+HIV‐Gag‐specific CD8+ T cells, promoting IFN‐γ secretion and enhancing cytotoxic potential, as indicated by CD107a expression [39]. These findings suggest a role for the IL‐27/STAT1 pathway in remodeling the effector functions of functionally exhausted CD8+ T cells.

In psoriasis, stimulation of human keratinocytes with IL‐27 results in significant upregulation of intracellular STAT1 phosphorylation. The resulting pSTAT1 subsequently binds to the promoter regions of specific chemokine genes, such as *CXCL9* and *CXCL10*, potently driving their expression and secretion [61, 86]. As classic IFN‐γ‐inducible chemokines, CXCL9 and CXCL10 efficiently recruit CXCR3+ effector T cells—notably Th1 and CD8+ T cells—to the site of cutaneous inflammation [61]. These infiltrating T cells then release large quantities of pro‐inflammatory cytokines, further amplifying the inflammatory cascade and leading to the formation of the characteristic skin lesions of psoriasis. Notably, recent research has identified a subset of CD8+ T cells with high granzyme K (GzmK) expression in patients with autoimmune diseases, such as inflammatory bowel disease and lupus nephritis. These cells not only secrete pro‐inflammatory cytokines but also utilize GzmK to directly stimulate synovial fibroblasts, inducing the production of IL‐6, CCL2, and reactive oxygen species (ROS) [87]. In Sjögren’s disease, GzmK disrupts mitochondrial integrity, causing the release of mitochondrial DNA, which triggers interferon production and tissue damage [88]. Whether IL‐27 regulates this non‐cytolytic subpopulation remains a critical knowledge gap.

Certainly, IL‐27 can also increase cytotoxicity via STAT‐independent signaling pathways, such as the AKT/mTOR pathway [89]. However, as the primary focus of this review is the IL‐27/STAT axis, pathways that do not directly involve STAT will not be discussed in further detail.

### 4.3. B Cells

The functional regulation of B cells by IL‐27 is also characterized by the complex interplay between the STAT1 and STAT3 signaling pathways, particularly with respect to antibody class‐switching and antigen‐presenting capacity. Murine in vitro studies have demonstrated that in B cells, the IL‐27/STAT1 signaling axis similarly upregulates *T-bet* expression, which in turn triggers class switching to IgG2a while concurrently inhibiting the production of IgG1 [90]. IgG2a confers crucial host protection against viral and parasitic pathogens; conversely, IL‐27 corrects Th2 skewing by inhibiting IL‐4‐induced IgG1 class switching, thereby restoring the Th1/Th2 immunoregulatory balance.

In multiple myeloma cells and plasma cells, IL‐27 predominantly activates STAT1, with only minor activation of STAT3, leading to the upregulation of the expression of the surface molecule CD38 [91]. This action thereby enhances the sensitivity of tumor cells to CD38‐targeted therapies.

Epstein–Barr virus (EBV)‐infected B cells express functional IL‐27 receptors. Both exogenously administered and autocrine IL‐27 can promote the growth and survival of these cells by activating STAT1 and STAT3, with the IL‐27/STAT1 axis being the principal driver of this effect [92]. In this regard, IL‐27 once again demonstrates its “double‐edged sword” role in terms of clinical manifestations. Initially, patients with IL‐27Rα deficiency experience severe acute primary infection upon first exposure to EBV. However, because the growth and proliferation of EBV‐infected B cells rely on autocrine IL‐27, and IL‐27Rα deficiency blocks this growth process, these patients recover well thereafter and do not develop chronic B‐cell lymphoproliferative diseases or lymphoma [92].

## 5. Functional Dynamic Balance of Innate Immune Cells

### 5.1. Monocytes and Macrophages

The regulation of monocytes and macrophages by the IL‐27/STAT axis is strongly bidirectional; depending on the microenvironment, the axis can amplify inflammatory responses while also exerting potent antiviral and antitumor effects.

Upon stimulation with IL‐27, human monocytes and macrophages upregulate a variety of pro‐inflammatory cytokines (including IL‐6, TNF‐α, and MIP‐1α) and chemokines (such as CXCL10) via the JAK1/2‐STAT1/3 pathway, thereby effectively amplifying the inflammatory response [93–96]. Specifically, pretreatment of monocytes with IL‐27 enhances their sensitivity and inflammatory reaction to subsequent LPS stimulation by upregulating TLR4 expression through the IL‐27/JAK2/STAT3 axis [94]. Furthermore, another study on human monocytes demonstrated that the IL‐27/STAT1 axis not only increases TLR sensitivity but also attenuates IL‐10 secretion [96]. Notably, however, there is a stark species‐specific difference in IL‐27 responsiveness. Unlike the robust response observed in human cells, murine bone marrow‐derived macrophages (BMDMs) respond minimally to IL‐27 stimulation, exhibiting no detectable phosphorylation of either STAT1 or STAT3 [96]. Accordingly, the weak or even absent response of BMDMs to IL‐27 suggests that murine models may not fully recapitulate the pro‐inflammatory signaling of IL‐27 observed in human macrophages. Therefore, caution is warranted when extrapolating findings from BMDMs to human biology.

In macrophages, the IL‐27/STAT1 axis concurrently upregulates MHC class II molecules to enhance the immune response and induces SOCS3 to mediate a negative feedback loop [93]. Furthermore, the IL‐27/STAT1 axis exerts its antiviral effect by inducing the expression of viral sensors (such as TLR3 and AIM2) and antiviral interferon‐stimulated genes (ISGs) (including MX1 and GBP5), thereby effectively inhibiting the replication of viruses such as Chikungunya virus (CHIKV) and Dengue virus (DENV‐2) [93]. Interestingly, while interferons can unidirectionally regulate the expression of IL‐27 subunits, the function of IL‐27 is not contingent upon IFN induction [93]. An in vitro study on healthy human macrophages revealed that the IL‐27‐JAK1/2‐STAT1/3‐TRIM axis (which involves TRIM19, 21, 22, and 69) can inhibit viral replication; the regulation of TRIM genes in this pathway induces innate immune and antiviral responses, including regulation of the viral life cycle [97]. During this process, the mechanism underlying the induction of antiviral proteins by IL‐27 is predominantly dependent on the STAT1‐IRF3 axis [97]. Furthermore, the IL‐27/STAT3 axis can induce the expansion of CD11b+Gr1+ myeloid cells and promote the infiltration of M1‐type macrophages into the tumor microenvironment, thereby exerting an antitumor effect [98].

Conversely, the anti‐inflammatory and cytoprotective effects mediated by the IL‐27/STAT axis in monocytes/macrophages have also been validated in numerous disease contexts. In lipopolysaccharide (LPS)‐stimulated murine microglia, by simultaneously activating STAT1 and STAT3, IL‐27 effectively triggers the production of the anti‐inflammatory cytokine IL‐10, thereby exerting a crucial immunomodulatory role that limits tissue damage from excessive inflammation [99]. The IL‐27/STAT1 axis can also inhibit the expression of the inflammatory mediator cyclooxygenase‐2 (COX‐2) and the generation of its product, prostaglandin E2 (PGE2), demonstrating a direct anti‐inflammatory effect of IL‐27 [100].

In tuberculosis, IL‐27 stimulates STAT3 phosphorylation in peritoneal macrophages to inhibit the release of key pro‐inflammatory cytokines, such as IL‐12 and TNF, thereby mitigating the inflammatory damage driven by these factors [101]. Within the specific pathological milieu of atherosclerosis, IL‐27‐mediated STAT3 activation has been shown to upregulate the expression of ATP‐binding cassette transporter A1 (ABCA1), which in turn inhibits foam cell formation and thereby lowers the risk of atherosclerotic development [102]. This highlights the cardiovascular protective potential of IL‐27. During the neonatal period, however, elevated levels of IL‐27, which acts in a STAT3‐dependent manner, impair the bacterial clearance capacity of macrophages, which to some extent exacerbates the clinical course of neonatal sepsis [103, 104]. Notably, neonatal mice with macrophage‐specific deletion of STAT3 exhibit heightened inflammatory responses and mortality rates, indicating that the IL‐27/STAT3 axis also serves a more critical protective function [104].

### 5.2. Dendritic Cells (DCs)

As professional antigen‐presenting cells, DCs play a central role in both the initiation of the adaptive immune response and the induction of immune tolerance. Genome‐wide analyses of monocyte‐derived human DCs have indicated that stimulation with IL‐27 significantly alters their miRNA expression profile [105].

IL‐27 signaling, through the activation of the JAK‐STAT pathway—specifically the key transcription factors STAT1 and STAT3—simultaneously programs dual functions of antiviral defense and immune tolerance within the same cell subset, thereby achieving a balance between infection control and tissue protection. In monocyte‐derived dendritic cells (MoDCs), IL‐27‐induced STAT1 phosphorylation is central to initiating this dual functionality. On one hand, IL‐27 significantly upregulates the expression of the coinhibitory molecule PD‐L1 (B7‐H1) on the surface of human MoDCs and myeloid DCs (mDCs) via the STAT1 signaling pathway, thereby inhibiting T‐cell proliferation and activation to induce immune tolerance [106]. On the other hand, within the same cell type, IL‐27 signaling also activates STAT1 to drive the expression of antiviral genes such as *MX1* and *OAS2* [107]. This process holds unique clinical significance as it effectively inhibits HIV‐1 replication at the reverse transcription stage via the phosphorylation of STAT1, STAT3, and STAT5, independent of Type I interferon involvement [107]. The co‐existence of PD‐L1 expression and antiviral gene expression on the same MoDCs reflects IL‐27’s strategy for programming DCs: endowing cells with intrinsic antiviral capabilities while simultaneously engaging the PD‐L1 checkpoint mechanism to prevent excessive inflammatory immune responses.

Beyond MoDCs, IL‐27‐mediated regulation of tolerance in other DC subsets also exhibits significant tissue specificity and mechanistic diversity. In conventional dendritic cells (cDCs), IL‐27 primarily relies on the STAT3 signaling axis to induce the upregulation of CD39 [108]. CD39 hydrolyzes pro‐inflammatory extracellular ATP, inhibiting P2X7 receptor‐mediated activation of the NLRP3 inflammasome, thereby metabolically limiting the release of cytokines such as IL‐1β [108]. Similarly, in plasmacytoid dendritic cells (pDCs) derived from the liver and spleen, IL‐27 demonstrates potent immunoregulatory capabilities [109]. Research indicates that IL‐27 upregulates B7‐H1 expression on the surface of these tissue‐resident pDCs via a STAT3‐dependent pathway, which is crucial for maintaining the immune‐tolerogenic microenvironment of organs such as the liver [109].

### 5.3. Natural Killer (NK) Cells

IL‐27 can potently activate natural killer (NK) cells, increasing both their cytotoxic activity and their capacity for cytokine secretion. Research has indicated that by synergistically activating the STAT1 and STAT3 signaling pathways, IL‐27 upregulates the expression of the key transcription factor *T-bet* and the release of GzmB, effects that collectively lead to increased IFN‐γ secretion and an enhanced capacity to kill target cells (such as tumor cells), thereby conferring dual pro‐ and anti‐inflammatory effects upon NK cells [110–112]. In a murine model of colon cancer, IL‐27 can induce and enhance NK cell‐mediated antitumor immunity via the activation of STAT3, a mechanism potentially involving the upregulation of perforin expression [113]. Conversely, in a murine model of liver cancer, the IL‐27/STAT axis has been shown to suppress the expression of cytotoxicity‐related genes (including *Gzmb*, Fas ligand, and *NKG2D*), thus promoting tumor growth and immune evasion [114].

Herein, the opposing roles of IL‐27 in colorectal cancer versus liver cancer underscore a critical paradigm: the functional outcome of IL‐27 is context‐dependent, dictated by the specific STAT signaling engaged within the tumor microenvironment. As observed in adaptive immunity, IL‐27 signaling is not monolithic. Reflecting the canonical signaling divergence described earlier, the tumor milieu biases the signaling balance toward either a cytotoxic STAT1‐driven program or a regulatory STAT3‐dominant state. Thus, IL‐27 acts as a molecular switch, wherein the differential engagement of these STATs to specific promoter sequences ultimately determines whether NK cells execute antitumor immunity or succumb to immune evasion.

### 5.4. Other Innate Immune Cells

In addition, the IL‐27/STAT axis influences other innate immune cell subsets. In the context of neuroinflammation following intracerebral hemorrhage, IL‐27 has a protective function: the IL‐27/STAT axis inhibits the expression of deleterious substances such as inducible nitric oxide synthase (iNOS) and matrix metalloproteinase‐9 (MMP‐9) in neutrophils, thereby mitigating inflammation‐induced cerebral edema and improving neurological function [115]. Macrophage‐derived IL‐27 activates the STAT1/STAT3 signaling pathway in microglia, dendritic cells, and astrocytes. This induces these glial cells to produce anti‐inflammatory proteins while reducing the release of pro‐inflammatory proteins and effectively inhibiting NLRP3 inflammasome activation, collectively maintaining immune homeostasis within the central nervous system (CNS) [116].

Conversely, in allergic diseases, the IL‐27/STAT1 axis predominantly exerts activating and pro‐inflammatory effects on eosinophils. This includes inhibiting their apoptosis, inducing the release of pro‐inflammatory cytokines (e.g., IL‐6 and IL‐1β) and chemokines (e.g., CCL2 and CXCL8), and upregulating surface adhesion molecules such as CD18 and ICAM‐1 to increase eosinophil accumulation at inflammatory sites [117]. Thus, within the context of allergic disease, IL‐27 exerts paradoxical effects on different cell populations: it drives a pro‐inflammatory function in eosinophils while simultaneously suppressing the Th2 cell‐induced inflammatory milieu.

## 6. IL‐27‐Mediated Immune Regulation Through Nonimmune Cells

The influence of IL‐27 on immune regulation via the JAK‐STAT pathway extends beyond classic immune cells to include a variety of nonimmune cell types, reflecting its complex and context‐dependent regulatory capacity. In these diverse cellular environments, IL‐27 signaling functions primarily through the modulation of cytokine and chemokine expression by nonimmune cells. These multifaceted roles highlight the broad biological relevance and pleiotropic nature of IL‐27 signaling, which extends well beyond its canonical functions in immune cells. Below, we systematically discuss these roles categorized by specific cell types.

### 6.1. Epithelial and Endothelial Cells

In epithelial and endothelial cells, IL‐27 displays opposing regulatory functions—acting as a pro‐inflammatory mediator in oral and vascular contexts while serving a protective role in the intestinal barrier. In oral epithelial cells, IL‐27 can activate the STAT1/3/Akt pathway to induce the production of Th1‐type cytokines (CXCL10 and CXCL11), a process that can be inhibited by honokiol and magnolol [118]. Similarly, during the early stages of transplant rejection (during ischemia‐reperfusion injury), IL‐27 exerts its pro‐inflammatory function by activating the STAT3 axis in endothelial cells, thus exacerbating the inflammatory response that culminates in rejection [119]. Furthermore, the IL‐27/STAT1 axis induces the sustained secretion of IL‐18 binding protein (BP) by human primary keratinocytes (HPKs), thereby exerting an anti‐inflammatory effect within the local cutaneous milieu through the antagonism of IL‐18 [120].

Conversely, IL‐27 confers significant protection in intestinal epithelial cells. Following oral administration of IL‐27 to mice with acute colitis, although levels of phosphorylated STAT1 were elevated in colonic mucosal epithelial cells and in inflammatory cells of the lamina propria and submucosa, this was paradoxically accompanied by a reduction in both the protein expression of pro‐inflammatory cytokines (such as IL‐6, IL‐1β, and TNF) and the extent of neutrophil infiltration [121]. Notably, in healthy, noncolitic mice, stimulation with IL‐27 did not result in a detectable STAT1 phosphorylation response [121]. In a different context, IL‐27 promoted the secretion of glucagon‐like peptide‐1 (GLP‐1) by activating a STAT3‐dependent mTOR signaling pathway in intestinal cells [122]. This IL‐27/STAT3/GLP‐1 axis can not only combat intestinal inflammation in inflammatory bowel disease but also stimulate insulin secretion and biosynthesis, positioning it as a promising therapeutic candidate for the amelioration of type 2 diabetes.

### 6.2. Nervous System Cells

**Figure 3:**
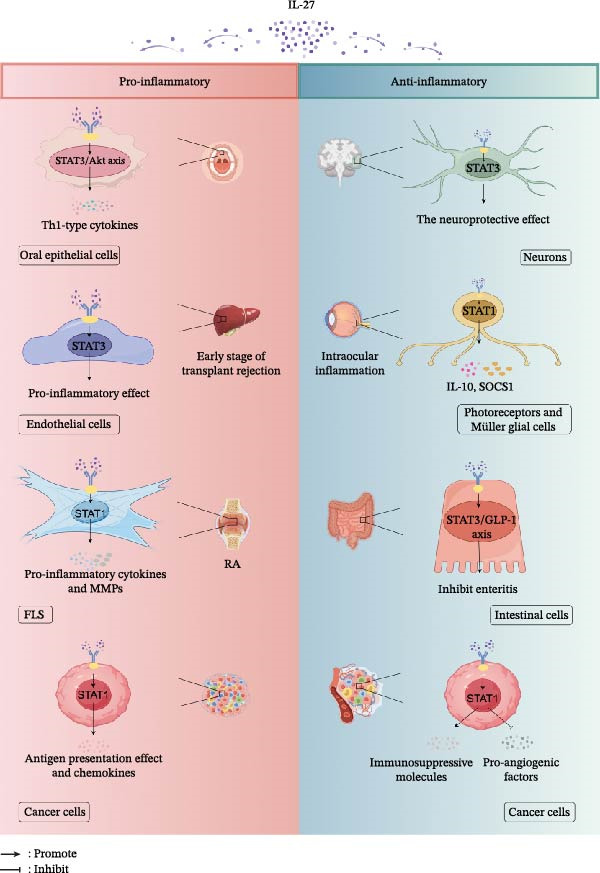
Schematic of IL‐27‐mediated immune regulation through JAK‐STAT signaling in nonimmune cells. This diagram highlights how IL‐27, by activating the JAK‐STAT signaling pathway, exerts opposing pro‐inflammatory and anti‐inflammatory/protective functions in various nonimmune cells. Pro‐inflammatory pathways (left) show that IL‐27 can stimulate oral epithelial cells to produce Th1‐type cytokines through the STAT3/Akt axis, drive fibroblast‐like synoviocytes (FLSs) to secrete pro‐inflammatory mediators and matrix metalloproteinases (MMPs) via the STAT1 axis, and enhance the antigen presentation and chemokine secretion effects of cancer cells. Conversely, the anti‐inflammatory pathways (right) indicate that IL‐27 can exert neuroprotective effects through STAT3 signaling in neurons, suppress intraocular inflammation via STAT1 signaling in retinal cells, inhibit enteritis through the STAT3/GLP‐1 axis in intestinal cells, and upregulate immunosuppressive molecules or inhibit proangiogenic factors in cancer cells via STAT1 signaling (created with Figdraw, https://www.figdraw.com). RA: rheumatoid arthritis; SOCS1: suppressor of cytokine signaling 1.

Within the central nervous system (CNS) and ocular tissues, IL‐27 predominantly exerts neuroprotective and anti‐inflammatory effects via the STAT1/STAT3 pathways (Figure 3). In a murine model of stroke, for example, the neuroprotective effect induced by IL‐27 was demonstrated to be contingent upon STAT3 signaling in neurons [123]. In models of intraocular inflammation, IL‐27 binds to its receptors on the surfaces of photoreceptors and Müller glial cells, which, in a STAT1‐dependent manner, induces the expression of IL‐10 and SOCS1. This helps to suppress excessive intraocular inflammation and protect the retina from damage [124].

### 6.3. Fibroblasts

While fibroblasts do not produce IL‐18, stimulation with IL‐27 activates intracellular STAT1 signaling to trigger the robust secretion of the IL‐18 antagonist, IL‐18 BP. This mechanism serves to suppress IL‐18‐driven chronic inflammation, such as that observed in psoriasis and cutaneous lupus erythematosus [120]. Notably, in the specific pathological microenvironment of RA, nonimmune stromal cells act as drivers of inflammation. In healthy joints, fibroblast‐like synoviocytes (FLS) maintain joint homeostasis and provide nutritional support by secreting hyaluronic acid and lubricin; however, in specific pathological conditions such as RA, FLS transform into “tumor‐like” cells that attack cartilage. IL‐27 preferentially activates STAT1 signaling in FLS. This significantly increases the expression of numerous pro‐inflammatory cytokines and MMPs, thereby driving chronic inflammation of the synovial tissue and progressive joint destruction [8] (Figure 3).

### 6.4. Cancer Cells

The IL‐27/STAT axis plays a complex, multilayered role in various cancer cells. It not only adds a layer of complexity by promoting immune evasion through the upregulation of immunosuppressive molecules but also contributes to anticancer mechanisms by enhancing tumor immunogenicity and inhibiting angiogenesis (Figure 3). The IL‐27/STAT1 axis can induce an IFN‐γ‐like response in cancer cells from diverse tissue origins (including human small‐cell lung cancer, hepatocellular carcinoma, melanoma, colon, and cervical cancer). This response is itself twofold: it can promote immune evasion by upregulating the expression of immunosuppressive molecules such as PD‐L1, yet it concurrently enhances tumor immunogenicity by upregulating the expression of antigen presentation‐related genes (such as the TAP transporter and MHC class I molecules) and chemokines (such as CXCL9 and CXCL11) [125, 126]. Additionally, via a STAT1‐dependent mechanism, IL‐27 can effectively inhibit the production of various proangiogenic factors, including IL‐8 and CXCL5 [127]. The suppression of these factors can, to some extent, inhibit neovascularization within lung cancer tissue, thereby restricting the tumor’s nutrient supply and growth [128].

## 7. Future Prospects

In this review, we highlight the “double‐edged sword” nature of IL‐27 signaling, in which immune outcomes are dictated by context‐dependent engagement of STAT1‐ versus STAT3‐dominated programs. This intrinsic duality poses a major translational challenge, as systemic enhancement or blockade of IL‐27 is unlikely to yield uniformly beneficial effects. For example, IL‐27 can simultaneously enhance CD8^+^ T‐cell cytotoxicity while promoting immune escape through STAT1‐dependent PD‐L1 induction on tumor or stromal cells. These observations underscore the need for more precise strategies to harness IL‐27 signaling.

One promising direction is the development of biased IL‐27 agonists that selectively favor specific STAT outputs. Recent structural insights into the IL‐27 quaternary receptor complex suggest several potential molecular entry points for such bias [9, 45, 129]. First, subtle modulation of the p28–IL‐27Rα binding interface could alter receptor geometry and JAK positioning, thereby skewing downstream STAT recruitment. Second, the distinct cytoplasmic phosphotyrosine motifs within IL‐27Rα and gp130, which differentially recruit STAT1 and STAT3, represent plausible targets for selectively reshaping STAT docking and signaling strength. Third, selective tuning of JAK1/JAK2 activation dynamics, informed by emerging allosteric JAK inhibitors, may allow preferential attenuation or preservation of specific STAT pathways without complete signal ablation.

Future studies integrating structural biology, single‐cell multiomics, and ligand engineering will be essential to decode how receptor architecture, intracellular STAT availability, and competing cytokine signals collectively determine IL‐27 function in vivo. Such efforts may enable the rational design of next‐generation IL‐27–based therapeutics that exploit its immunoregulatory potential while minimizing pathogenic signaling.

## Author Contributions

Xiangqi Zhang designed and wrote the manuscript. Hedong Zhang revised the manuscript. Chen Feng, Ye Xu, and Yingqi Zeng reviewed the manuscript. Qiulin Luo, Jiajie Qin, Tengfang Li, and Longkai Peng conducted the literature search and assisted in revising the manuscript. Helong Dai contributed to the concept and outline of manuscript, proofread, and supervised, and the final approval of the version to be published.

## Funding

This work was supported by the National Natural Science Foundation of China (Grants 82270796 and 82470789 to Helong Dai, 82200849 to Tengfang Li, and 82370761 to Longkai Peng), the Science and Technology Innovation Program of Hunan Province (Grant 2022RC3071 to Helong Dai), National Science and Technology Major Project of China (2025ZD0552200 to Helong Dai), Strategic Research and Consulting Project of Hunan Institute of Engineering Technology Development Strategy in China (Grant 2025WK006 to Helong Dai).

## Disclosure

All authors have read and approved the final manuscript.

## Conflicts of Interest

The authors declare no conflicts of interest.

## Data Availability

Data sharing is not applicable to this article as no datasets were generated or analyzed during the current study.
